# Un carcinome épidermoïde du col utérin récidivant sous forme d'une métastase pulmonaire isolée: à propos d'un cas et revue de la literature

**DOI:** 10.11604/pamj.2015.22.369.7217

**Published:** 2015-12-16

**Authors:** Rajae Kanab, Fazoumed El M'rabet, Taoufiq Ameuraoui, Fatoumata Sidibé, Siham Tizniti, Afaf Amarti, Samia Arifi, Nawfal Mellas

**Affiliations:** 1 Service d'Oncologie Médicale, CHU Hassan II, Fès, Maroc; 2 Service de Radiologie, CHU Hassan II, Fès, Maroc; 3 Laboratoire d'Anatomopathologie Al Azhar, Fès, Maroc

**Keywords:** Carcinome épidermoïde, col utérin, métastase, poumon, p16, Squamous cell carcinoma, cervix, metastasis, lung, p16

## Abstract

Les métastases pulmonaires du carcinome épidermoïde (CE) du col utérin sont rares. C'est un cancer à évolution locorégionale, rarement extra pelvienne, et qui est intimement lié à l'infection par HPV (humain papilloma virus). Nous rapportons un nouveau cas d'une patiente âgée de 40 ans, traitée pour CE du col utérin il y a 3 ans, traitées par RTH externe, curiethérapie et chirurgie et qui présente une récidive de sa maladie sous forme d'une masse pulmonaire isolée mimant un primitif pulmonaire. Le diagnostic a été posé par génotypage HPV sur le prélèvement biopsique de la masse pulmonaire. La patiente a été mise sous chimiothérapie type col utérin, avec une bonne réponse clinique et biologique.

## Introduction

Le cancer du col utérin est le deuxième cancer chez la femme après le cancer du sein dans le monde. C'est une maladie liée essentiellement à l'infection par HPV (humain papilloma virus) dans 89%des cas [1]. Son incidence au Maroc est de 2000 nouveaux cas par an [2]. Le cancer du col utérin est à évolution locorégionale, les métastase extra pelviennes sont rares notamment au niveau du poumon. Les métastases pulmonaires du cancer du col utérin ont été rapportées chez 3,1%-9,9% des cas [3]. Le diagnostic précis de métastases pulmonaires d'un cancer du col utérin reste difficile vue la rareté de cet événement et l'absence de facteurs de risques prédisposant reconnus. Le génotypage HPV peut aider à la discrimination entre un primitif pulmonaire et des métastases d'origine du col utérin [1].

## Patient et observation

Les auteurs déclarent d'avoir pris le consentement de la patiente pour publier ses renseignements cliniques. Mme F. A âgée de 39ans, mariée et mère de 6 enfants. Suivie depuis 2001 pour un carcinome CE du col utérin classé T1b avec un bilan d'extension normal; la patiente a reçu une chimio-radiothérapie pré-opératoire à la dose de 45Gy, suivie d'une curiethérapie utéro-vaginale à la dose de 25Gy, avec une régression partielle de la tumeur, par la suite elle a été opérée, une colpohysterectomie totale sans curage. L'examen anatomo-pathologique de la pièce opératoire n'a pas montré de résidu tumoral, les collerettes vaginales, paramètres et annexes étaient indemnes d’élément tumoral.

**Figure 1:**
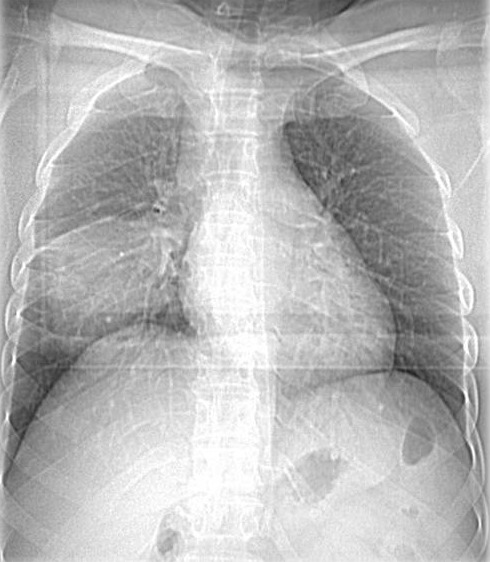
Radio thorax: opacité LIDt de contours flous à limite supérieur scissurale nette

**Figure 2:**
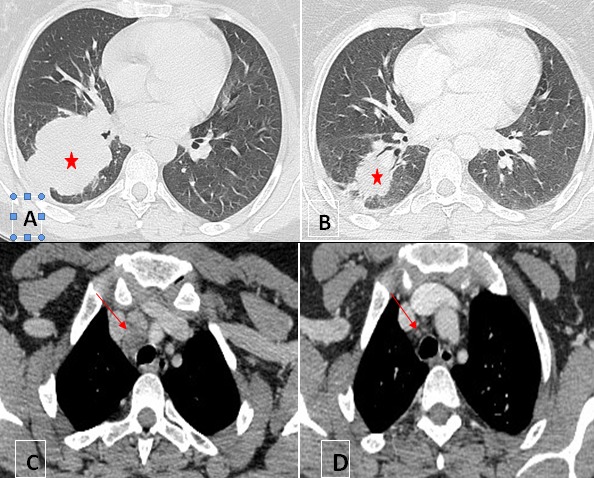
Coupes axiales tomodensitomériques (TDM) en fenêtre parenchymateuse. (A) masse tumorale parenchymateuse pulmonaire LID unique mesurant 08cm de grand axe avec extension hilaire et pleurale homolatérales; (B) contrôle après chimiothérapie: nette régression de la masse mesurant 04cm de grand axe. Coupes axiales TDM en fenêtre médiastinale: (C) adénopathies médiastinales de la loge de barety centimétriques; (D) contrôle après chimiothérapie: ganglions médiastinaux de la loge de baretyinfracentimétriques

Après 3 ans de suivi régulier la patiente s'est présentée à la consultation avec une histoire de toux sèche et amaigrissement depuis 4 mois; une radio thorax standard (Figure 1) a été demandée et qui a révélé la présence d′une opacité (lobaire inférieure droite) LIDt de contours flous à limite supérieur scissurale nette; on a complété par une TDM Thoracique (Figure 2 A et C) qui a montré la présence d'une masse tissulaire pulmonaire lobaire inférieure droite, hétérogène avant et après injection de produit de contraste, de contour irréguliers mesurant 70*60*68 mm, englobant les branches segmentaires artérielles de la lobaire inférieure ainsi que les bronches segmentaires aériques, lobaires inférieurs. Avec quelques adénopathies médiastinales de la loge de Barety, pré et sous carinaires, hilaires droites pour la plus grande 18 mm. On a complété parune TDM abdominale et IRM pelvienne qui n'ont pas révélé d'autres lésions secondaires ou de récidive locale.

**Figure 3:**
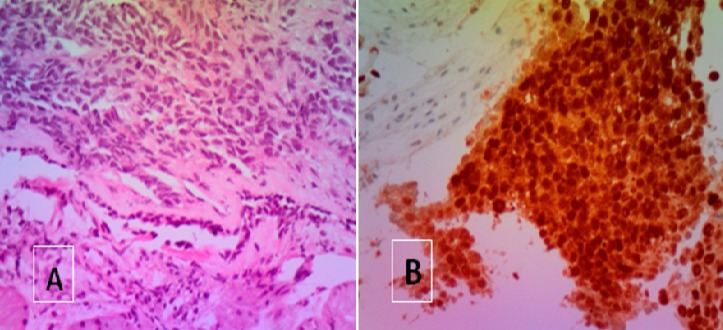
Métastase pulmonaire d'un processus carcinomateux peu différencié HE x200 B: forte expression nucléaire de la protéine p16 au niveau de la métastase pulmonaire X200

La patiente a eu une biopsie scanno-guidée du nodule pulmonaire et l étude anatopatholgique est revenue en faveur d'un processus carcinomateux TTF1 négatif, p63 négatif et p16 fortement positif compatible avec une origine cervicale (Figure 3 A et B). La patiente a été mise sous chimiothérapie type col utérin: CDDP 50mg/m^2^ et paclitaxel 175 mg/m^2^ avec une bonne réponse clinique et radiologique (Figure 2 B et D).

## Discussion

La récidive pulmonaire isolée du carcinome épidermoide du col utérin est rare. Les localisations qui donnent plus fréquemment des métastases pulmonaires dans l'ordre de fréquence sont: le sein, le larynx, la prostate, lathyroïde, la vessie, l'estomac et le pancréas [4]. Le cancer du col se propage plus fréquemment par extension directe aux tissus de voisinage comme le vagin, l′utérus, et les organes de la cavité pelvienne.

Les métastases extra pelviennes sont rencontrées dans les stades avancées de la maladie tels que les poumons, les ganglions lymphatiques para-aortiques et les os. Les métastases au poumon comprennent jusqu′à 3% d′échecs thérapeutiques du cancer du col de stade IA, 15% au stade IB, 20-25% au stade IIB, et 40% au stade IIB [5]. L'incidence des métastase pulmonaires au cours du cancer du col utérin diffère selon le type histologique de la tumeur. Le risque est accru chez les patientes avec: un adénocarcinome, le cancer anaplasique du col utérin, et les tumeurs neuroendocrines à petites cellules. Les métastases du carcinome épidermoïde du col sont moins fréquentes et habituellement ne dépassent pas 5% [6].

Dans ce contexte, p16 est un marqueur utile pour la discrimination entre les carcinomes épidermoïde du col de l′utérus et pulmonaire comme une surexpression de p16 a été constamment observée dans le cancer du col HPV lié. La protéine p16INK4a est induite dans les cellules basales de l’épithélium malpighien du col utérin par l'expression des oncogènes viraux E6 et E7 au cours d'une infection à HPV à haut risque [7, 8], d'où l'intérêt de la recherche de cette anti corps pour différencier entre un carcinome pulmonaire primitif et secondaire du col. Chez notre patiente la sur expression de p16 a été recherchée sur la tumeur primitive et la métastase pulmonaire, et elle est revenue positive sur les deux.

Dans la littérature les manifestations cliniques des métastases pulmonaires tels que la dyspnée et la toux non productive conduisent souvent à un diagnostic incorrect de la pneumonie, l′embolie pulmonaire, l′insuffisance cardiaque congestive, l′asthme, et la sarcoïdose [9], dans notre cas le mode de révélation était une dyspnée avec toux sèche ce qui a conduit à tord à un diagnostic de pneumonie, devant la résistante au traitement les d'autres examens ont été demandées notamment la biopsie de la lésion pulmonaire. L’étiologie de la récidive tumorale au niveau pulmonaire exclusivement et les facteurs qui lui prédisposent sont complexes, rares, et pas entièrement compris.

## Conclusion

Les métastases pulmonaires isolées du carcinome épidermoïde du col utérin sont rares, et leurs mécanisme, n'est pas bien élucidé. La recherche de l'expression de p16 sur la métastase et la tumeur primitive confirme le diagnostic et écarte la possibilité d'un primitif pulmonaire.
